# Effect of Socioeconomic Deprivation and Health Service Utilisation on Antepartum and Intrapartum Stillbirth: Population Cohort Study from Rural Ghana

**DOI:** 10.1371/journal.pone.0039050

**Published:** 2012-07-13

**Authors:** Yoonhee P. Ha, Lisa S. Hurt, Charlotte Tawiah-Agyemang, Betty R. Kirkwood, Karen M. Edmond

**Affiliations:** 1 Faculty of Epidemiology and Population Health, London School of Hygiene and Tropical Medicine, London, United Kingdom; 2 Kintampo Health Research Centre, Kintampo, Brong Ahafo, Ghana; 3 School of Paediatrics and Child Health, University of Western Australia, Perth, Western Australia, Australia; University of Cape Town, South Africa

## Abstract

**Background:**

No studies have examined the effect of socioeconomic deprivation on antepartum and intrapartum stillbirths in the poorest women in low income countries.

**Methodology/ Principal Findings:**

This study used data from a prospective population based surveillance system involving all women of childbearing age and their babies in rural Ghana. The primary objective was to evaluate associations between household wealth and risk of antepartum and intrapartum stillbirth. The secondary objective was to assess whether any differences in risk were mediated by utilisation of health services during pregnancy. Data were analysed using multivariable logistic regression. Random effect models adjusted for clustering of women who delivered more than one infant. There were 80267 babies delivered from 1 July 2003 to 30 September 2008: 77666 live births and 2601 stillbirths. Of the stillbirths 1367 (52.6%) were antepartum, 989 (38.0%) were intrapartum and 245 (9.4%) had no data on the timing of death. 94.8% of the babies born in the study (76129/80267) had complete data on all covariates and outcomes. 36 878 (48.4%) of babies were born to women in the two poorest quintiles and 3697 (4.9%) had no pregnancy care. There was no association between wealth and antepartum stillbirths. There was a marked ‘dose response’ of increasing risk of intrapartum stillbirth with increasing levels of socioeconomic deprivation (adjOR 1.09 [1.03–1.16] p value 0.002). Women in the poorest two quintiles had greater risk of intrapartum stillbirth (adjOR 1.19 [1.02–1.38] p value 0.023) compared to the richest women. Adjusting for heath service utilisation and other variables did not alter results.

**Conclusions/ Significance:**

Poor women had a high risk of intrapartum stillbirth and this risk was not influenced by health service utilisation. Health system strengthening is required to meet the needs of poor women in our study population.

## Introduction

Over 98% of stillbirths occur in low and middle income countries and sub-Saharan Africa has the greatest risk [1]. There are marked disparities in stillbirth risk in high income countries and the highest numbers are found in the deprived and marginalised groups in these countries [2]–[5]. Recent publications have assessed aetiology and interventions to reduce stillbirth in low and middle income countries [1], [6], [7]. However, no published studies have examined differentials in antepartum and intrapartum stillbirth risk in poor countries. Analyses from the Macro International Demographic and Health Surveys (DHS) are limited by small sample sizes and underreporting [8]. These surveys also do not differentiate between antepartum and intrapartum stillbirths despite their substantially different determinants and pathogenesis [8].

There is much evidence about the impact of delivery care on perinatal outcomes [1], [9]–[11]. However, there is less information about the effect of health service utilisation during pregnancy on perinatal outcomes, especially in low income countries [12]. In particular, there have been no published studies which have examined the effect of health service utilisation during pregnancy on perinatal outcomes in the poorest women. This information is needed to ensure that antenatal care programmes are implemented and targeted appropriately and to understand whether health services are reaching the women who are most in need.

A large prospective population based surveillance system was developed in rural Ghana for the ObaapaVitA trial, a cluster-randomised, double-blind, placebo-controlled trial to assess the effect of vitamin A supplementation on maternal survival [13]. Our study was designed to analyse data obtained from this surveillance system. The primary objective was to determine whether the risk of antepartum and intrapartum stillbirth differed by maternal wealth quintile in rural Africa. The secondary objective was to assess whether any differences in risk were mediated by utilisation of health services during pregnancy.

## Methods

### Study setting

Data for this study were collected from 1 July 2003 to 30 September 2008 as part of the ObaapaVitA trial which was implemented in seven contiguous rural districts in the Brong Ahafo region of Ghana. All women of reproductive age (15–45 years) who provided informed written consent and intended to live in the study area for at least three months were eligible to enrol in the trial. The full protocol is available online (at http://www.lshtm.ac.uk/eph/nphir/research/obaapavita/obaapavita_trial_protocol.pdf).

Pregnancy care in Ghana includes education about the importance of birth preparedness, danger signs, care seeking and facility delivery. Women are also examined for obstetric problems such as placental insufficiency and multiple pregnancy. Tetanus toxoid vaccination, iron and folate supplementation, and screening for malnutrition and infection is also included [8]. It is recommended that each woman visits a health care provider at least four times even if the pregnancy is uncomplicated. Pregnancy care is provided health facilities (district hospitals, health centres and health posts) and there are no outreach services [8].

### Data collection

All women of reproductive age were visited once every four weeks by a network of trained village-based fieldworkers to distribute vitamin A capsules and collect data on morbidity and mortality. During pregnancy the fieldworkers administered a “profile” questionnaire which recorded the woman's self report of sociodemographic characteristics (maternal age, ethnic group, education, religion, asset ownership), and past obstetric history (previous stillbirth, gravidity). When a delivery was reported, the fieldworker also administered a “birth” questionnaire, which included the mother's self report of delivery outcome (live birth or infant not born alive), site and mode of delivery, health service utilisation during the pregnancy (number of visits to a health care provider during pregnancy), and details about the infant (multiple or singleton birth, gender, gestational age and birth weight if recorded on the pregnancy or birth record). Data on quality of pregnancy care were also recorded, however these data were especially limited by maternal recall. Data were only available on one measure of the quality of pregnancy care (number of tetanus toxoid immunisations received up to the time of delivery).

Field supervisors conducted verbal post-mortems (VPMs) with the mother and close family members for all deliveries which resulted in a dead fetus or infant. Information collected included an open history plus questions on any signs, symptoms and illnesses during pregnancy, delivery and after delivery. Mothers were specifically asked when fetal movements ceased i.e. before labour or during labour/delivery. Standard World Health Organization VPM tools and methods were used. The methods have been presented and validated in earlier papers [14], [15].

### Definitions and classification

Delivery outcomes were classified based on the information obtained from the VPM interview [15]. The VPMs were reviewed by two experienced physicians, who independently decided on gestation, timing and cause of death. A miscarriage/abortion was defined as delivery which ended in a dead foetus or infant at <6 months gestation. A stillbirth was defined as an infant of ≥6 months of gestation who did not cry, move, or breathe after birth. Antepartum stillbirths were classified when the infant died before labour commenced. Intrapartum stillbirths were classified when the infant died during labour or delivery. Neonatal deaths were classified if the infant was born alive but died day 0–27. Postneonatal deaths were classified if the infant was born alive but died day 28–365. If the physicians disagreed, the form was independently reviewed by a third physician and a consensus coding accepted if two of the three agreed. If there was no consensus, the three physicians met to reach an agreement.

To estimate the socioeconomic status of each household, we constructed a relative wealth index based on data collected on housing material (walls, floor, windows, and roof) and household assets. Principal component analysis (PCA) in STATA version 11 was used to generate a factor score for each asset and women were assigned to wealth quintiles according to their total asset score [16]. The wealth quintile variable was examined as categorical and continuous in the multivariable models, and we also created a binary variable in which women in the lowest two quintiles (“poor”) and in the top three quintiles (“richer”) were grouped.

Health service utilisation during pregnancy (pregnancy care) was examined using data on the number of visits to a health care provider during pregnancy. Pregnancy care was defined as the number of visits to a health care provider during pregnancy but before delivery (what is commonly called antenatal care) and did not include care during childbirth. We decided not to use the term ‘antenatal’ to avoid confusion with the words used to describe our primary outcomes (antepartum and intrapartum stillbirths). Pregnancy care was examined as a binary variable (no care versus some care) and as a continuous variable (based on the number of visits). We decided a priori not to examine site of delivery or care during labour or delivery because we felt that care seeking at this time would be strongly influenced by maternal morbidity and obstetric complications. We felt that women who are having a complicated pregnancy would be more likely to seek care from a hospital than a woman who is having an uncomplicated pregnancy and that this would result in higher intrapartum stillbirth risks in district hospitals compared to health centres or women delivering at home.

### Statistical analysis

All deliveries (live births and stillbirths) to women enrolled in the surveillance system between 1 July 2003 and 30 September 2008 were eligible for inclusion in the analysis. Deliveries with missing exposure or outcome data and delivery outcomes with no agreed gestation or timing of death were excluded from all analyses.

Logistic regression was used to examine the effect of maternal wealth quintile on antepartum and intrapartum stillbirth separately. Odds ratios (OR) and 95% confidence intervals (95% CI) were used to approximate relative risks of antepartum and intrapartum stillbirth. Multivariable logistic regression models were used to assess the effect of important explanatory variables and random effects models were used to adjust for potential clustering of outcomes in mothers with more than one delivery over the study period. The first multivariable model examined the effect of wealth quintile adjusted only for clustering by woman. The second model included wealth quintile and the key variable relating to maternal health service utilisation (number of visits for pregnancy care). Maternal sociodemographic characteristics (maternal age, ethnic group, education), past obstetric history (previous stillbirth, gravidity), infant factors (sex, multiple or singleton birth) and maternal health service utilisation (number of visits for pregnancy care) were included a priori in the third model. We also examined statistical interactions between wealth and health service utilisation and hypothesised a priori that the effect of health service utilisation on antepartum and intrapartum stillbirths might be different in rich and poor women. Likelihood ratio tests were used to compare the fit of models containing different variables, to test for departures from a linear trend in ordered categorical variables and to test for a statistical interaction between wealth and health service utilisation.

### Ethical considerations

Ethical approval for the study was received from the ethics committees of the Ghana Health Service and the London School of Hygiene & Tropical Medicine. Full, written informed consent was obtained from all women who participated in this study. Full, written informed consent was also obtained from all mothers of infants involved in the study. If the mother was not available full written informed consent was obtained from the primary caretaker of the infant.

## Results

### Study population

There were 80267 babies born (77666 live births and 2601 stillbirths) to 63709 women from 1 July 2003 to 30 September 2008. Of these, 2177 were neonatal deaths (neonatal mortality risk 28.0/1000 live births) and 2601 were stillbirths (stillbirth risk 32.4/1000 live and still births). There were 1367 (52.6%) antepartum stillbirths, 989 (38.0%) intrapartum stillbirths and 245 (9.4%) stillbirths did not have data on timing of death.76129 babies born in the study (94.8%) had complete data on all covariates and outcomes (Table 1). 36878 (48.4%) babies were born to women in the two poorest quintiles. 3959 (5.2%) babies were born to women who had delivered a previous stillbirth. 8146 (10.7%) were born to women under 20 years of age and 9440 (12.4%) were to primiparas. 3350 (4.4%) were multiple births. Recorded birthweight from the pregnancy card was only available for 40.3% (30 680) of all births and 12.0% (9135) of stillbirths. 33040 (43.4%) babies were born at home and 3697 (4.9%) had no pregnancy care (Table 1). Women in our study population could only provide limited information about the quality of care that they had received in health care facilities. Only 29687 women (41%) were able to provide a response to questioning about the number of tetanus toxoid vaccinations received by the end of pregnancy. 25357 (85.4%) of the responders reported receiving two or more tetanus toxoid vaccinations by the time of delivery.

**Table 1:** Maternal characteristics and antepartum and intrapartum stillbirths, 1 July 2003 to 30 September 2008.

	Live births n (%) n = 77 666	Antepartum stillbirths n (%) n = 1367	Intrapartum stillbirths n (%) n = 989
**Wealth quintile**			
Highest (richest)	11 578 (14.9)	216 (15.8)	119 (12.0)
Second	13187 (17.0)	240 (17.6)	149 (15.1)
Middle	14 590 (18.8)	253 (18.5)	187 (18.9)
Fourth	16 869 (21.7)	312 (22.8)	240 (24.3)
Lowest (poorest)	19 818 (25.5)	314 (23.0)	267 (27.0)
Data missing (%)	1624 (2.1)	32 (2.3)	27 (2.7)
**Number of visits for pregnancy care**			
0	3696 (4.8)	103 (7.5)	50 (5.1)
1	5692 (7.3)	83 (6.1)	72 (7.3)
2–3	19147 (24.7)	331 (24.2)	246 (24.9)
≥4	47 079 (60.6)	802 (58.7)	575 (58.1)
Data missing (%)	2052 (2.6)	48 (3.5)	46 (4.7)
**Site of delivery**			
Home	33780 (43.5)	401 (29.3)	329 (33.3)
Private health centre	4944 (6.4)	48 (3.5)	27 (2.7)
Government health centre	16722 (21.5)	207 (15.1)	168 (17.0)
District or regional hospital	22118 (28.5)	688 (50.3)	459 (46.4)
Data missing (%)	102 (0.1)	23 (1.7)	6 (0.6)
**Highest educational level**			
None	29 379 (37.8)	506 (37.0)	411 (41.6)
Primary school	15 160 (19.5)	264 (19.3)	183 (18.5)
Secondary school or higher	32 711 (42.1)	578 (42.3)	386 (39.0)
Data missing (%)	416 (0.5)	19 (1.4)	9 (0.9)
**Age (years)**			
<20	8313 (10.7)	147 (10.8)	87 (8.8)
20–24	20763 (26.7)	332 (24.3)	265 (26.8)
25–29	21 429 (27.6)	324 (23.7)	238 (24.1)
30–34	15 330 (19.7)	271 (19.8)	218 (22.0)
35–39	8242 (10.6)	195 (14.3)	121 (12.2)
>40	3589 (4.6)	98 (7.2)	60 (6.1)
Data missing (%)	0 (0.0)	0 (0.0)	0 (0.0)
**Ethnic group**			
Akan	33892 (43.6)	672 (49.2)	405 (41.0)
Other	43407 (55.9)	677 (49.5)	575 (58.1)
Data missing (%)	367 (0.5)	18 (1.3)	9 (0.9)
**Gravidity**			
0	9624 (12.4)	199 (14.6)	125 (12.6)
1	15178 (19.5)	276 (20.2)	195 (19.7)
2	13 932 (17.9)	161 (11.8)	139 (14.1)
≥3	38 720 (49.9)	715 (52.3)	522 (52.8)
Data missing (%)	212 (0.3)	16 (1.2)	8 (0.8)
**Previous stillbirth**			
0	74171 (95.5)	872 (63.8)	682 (69.0)
≥1	3293 (4.2)	479 (35.0)	299 (30.2)
Data missing (%)	202 (0.3)	16 (1.2)	8 (0.8)
**Sex of infant**			
Female	38342 (49.4)	545 (39.8)	391 (39.5)
Male	39265 (50.6)	726 (53.1)	575 (58.1)
Data missing (%)	59 (0.1)	96 (7.0)	23 (2.3)
**Number of infants delivered**			
1	74 446 (95.8)	1272 (93.1)	823 (83.2)
>1	3220 (4.2)	95 (6.9)	166 (16.8)
Data missing (%)	0 (0.0)	0 (0.0)	0 (0.0)

Stillbirths for which timing of death was unknown are not included.

### Maternal sociodemographics and obstetric history

The strongest predictor of antepartum stillbirths was experiencing a stillbirth in a previous pregnancy (adjusted odds ratio [adjOR] 14.12,95% confidence interval [12.27–16.24], p value <0.001). Antepartum stillbirths were also more likely in older women, in Akan women, in women having their first or second births, where the child was male, or in multiple births (Table 2). Similar patterns were seen for intrapartum stillbirths, although ethnic group were not significantly associated with this type of stillbirth. A previous stillbirth was also the strongest predictor of intrapartum stillbirth, although births from a multiple pregnancy were almost five times more likely to end in an intrapartum stillbirth (adjOR 4.62 [3.79–5.64] p value <0.001) (Table 2).

**Table 2:** Associations between stillbirths, maternal sociodemographic characteristics and obstetric history, 1 July 2003 to 30 September 2008.

	Antepartum stillbirths		Intrapartum stillbirths	
	Univariable OR (95% CI)	Multivariable* OR (95% CI)	Univariable OR (95% CI)	Multivariable* OR (95% CI)
**Number of visits for pregnancy care**				
Any	1.00	1.00	1.00	1.00
None	1.37 (1.07, 1.76)	1.64 (1.27, 2.10)	1.01 (0.73, 1.39)	1.09 (0.79, 1.50)
P value	0.01	<0.001	0.95	0.62
**Site of delivery**				
Home	1.00	1.00	1.00	1.00
Private health centre	0.83 (0.60, 1.14)	0.93 (0.67, 1.29)	0.54 (0.35, 0.82)	0.66 (0.43, 1.01)
Government health centre	1.00 (0.83, 1.21)	1.03 (0.85, 1.25)	1.01 (0.82, 1.24)	1.07 (0.87, 1.32)
District or regional hospital	2.68 (2.33, 3.08)	2.59 (2.21, 3.03)	2.13 (1.83, 2.50)	2.22 (1.86, 2.64)
P value	< 0.001	< 0.001	<0.001	<0.001
**Highest educational level**				
None	1.00 (0.85, 1.18)	1.11 (0.94, 1.30)	1.06 (0.88, 1.28)	1.05 (0.86, 1.28)
Primary school	0.99 (0.86, 1.13)	1.17 (0.99, 1.38)	1.17 (1.00, 1.36)	1.08 (0.89, 1.30)
≥Secondary school	1.00	1.00	1.00	1.00
P value	0.98	0.17	0.14	0.75
**Age (years)**				
<20	1.24 (1.00, 1.54)	0.95 (0.75, 1.19)	0.95 (0.73, 1.24)	0.79 (0.59, 1.05)
20–24	1.11 (0.93, 1.31)	0.97 (0.81, 1.15)	1.16 (0.96, 1.41)	1.04 (0.85, 1.27)
25–29	1.00	1.00	1.00	1.00
30–34	1.10 (0.91, 1.32)	1.15 (0.96, 1.39)	1.31 (1.07, 1.60)	1.41 (1.15, 1.72)
35–39	1.60 (1.30, 1.96)	1.56 (1.27, 1.92)	1.24 (0.97, 1.59)	1.25 (0.97, 1.61)
≥40	1.78 (1.36, 2.32)	1.64 (1.26, 2.15)	1.58 (1.16, 2.15)	1.54 (1.13, 2.12)
P value	<0.001	<0.001	0.01	<0.001
**Ethnic group**				
Akan	1.00	1.00	1.00	1.00
Other	0.81 (0.72, 0.91)	0.81 (0.70, 0.93)	1.08 (0.94, 1.24)	1.01 (0.86, 1.20)
Pvalue	0.001	0.003	0.29	0.86
**Gravidity**				
0	1.93 (1.53, 2.43)	2.84 (2.24, 3.61)	1.28 (0.98, 1.66)	1.91 (1.45, 2.51)
1	1.63 (1.32, 2.03)	1.87 (1.51, 2.32)	1.30 (1.03, 1.64)	1.53 (1.21, 1.94)
2	1.00	1.00	1.00	1.00
≥3	1.58 (1.31, 1.92)	0.95 (0.77, 1.16)	1.31 (1.07, 1.61)	0.81 (0.65, 1.00)
P value	<0.001	<0.001	0.05	<0.001
**Previous stillbirth**				
0	1.00	1.00	1.00	1.00
≥1	11.59 (10.20, 13.18)	14.12 (12.27, 16.24)	9.02 (7.71, 10.56)	9.97 (8.44, 11.77)
P value	<0.001	<0.001	<0.001	<0.001
**Sex of infant**				
Female	1.00	1.00	1.00	1.00
Male	1.29 (1.14, 1.46)	1.25 (1.11, 1.40)	1.48 (1.29, 1.71)	1.43 (1.25, 1.64)
P value	<0.001	<0.001	<0.001	<0.001
**Number of infants delivered**				
1	1.00	1.00	1.00	1.00
>1	1.63 (1.27, 2.10)	1.56 (1.23, 1.99)	4.89 (3.98, 6.01)	4.62 (3.79, 5.64)
P value	<0.001	<0.001	<0.001	<0.001

Analyses conducted on mothers and infants with no missing data (n = 76 129).

* Multivariable analyses adjusted for wealth quintile, health service utilisation (number of visits for pregnancy care), maternal age, ethnic group, gravidity, previous stillbirth, number of infants delivered, sex of the infant, clustering by woman.

### Health service utilisation

Antepartum stillbirths were more likely where the woman had no care during the pregnancy (adjOR 1.64 [1.27–2.10], p value <0.001); however, pregnancy care was not associated with intrapartum stillbirths in the univariable or multivariable models (Table 2). Women in the poorest quintile were more likely to have no care in the current pregnancy than women in the richest quintile (adjOR 16.22 [12.19–21.59], p value <0.001) (Table 3). Women in the poorest quintile were also more likely to have received poor quality pregnancy care (<2 tetanus toxoid immunisations) than the women in the richest quintile (adjOR 3.47 [2.85–4.22], p value <0.001). Women in the poorest quintile were also more likely to deliver at home than women in the richest quintile (adjOR 17.98 [11.82, 23.90], p value <0.001) (Table 3).

**Table 3:** Health service utilisation and quality by maternal socioeconomic status, 1 July 2003 to 30 September 2008.

	Highest (richest)	Second	Middle	Fourth	Lowest (poorest)	Total
**Quality of pregnancy care**						
<2 doses tetanus toxoid	321 (6.5%)	590 (11.0%)	692 (11.5%)	1144 (17.2%)	1583 (23.5%)	4330 (14.6%)
≥2 doses tetanus toxoid	4596 (93.5%)	4779 (89.0%)	5312 (88.5%)	5516 (82.8%)	5154 (76.5%)	25 357 (85.4%)
Total	4917 (100%)	5369 (100%)	6004 (100%)	6660 (100%)	6737 (100%)	29 687 (100%)
Adjusted odds ratio (95%CI)*	1.00	1.88 (1.55, 2.27)	1.90 (1.57, 2.29)	2.77 (2.29, 3.34)	3.47 (2.85, 4.22)	
P value	-	<0.001	<0.001	<0.001	<0.001	
**Number of visits for pregnancy care**						
0	75 (0.7%)	180 (1.4%)	397 (2.7%)	856 (5.0%)	2189 (11.0%)	3697 (4.9%)
1	164 (1.4%)	534 (4.1%)	813 (5.6%)	1535 (9.0%)	2637 (13.2%)	5683 (7.5%)
2–3	1083 (9.4%)	2286 (17.4%)	3415 (23.4%)	5356 (31.6%)	7155 (35.9%)	19 295 (25.3%)
≥4	10 201 (88.5%)	10 148 (77.2%)	9955 (68.3%)	9218 (54.3%)	7932 (39.8%)	47 454 (62.3%)
Total	11 523 (100%)	13 148 (100%)	14 580 (100%)	16 965 (100%)	19 913 (100%)	76 129 (100%)
Adjusted odds ratio (95%CI)**	1.00	1.99 (1.46, 2.72)	4.26 (3.18, 5.69)	7.11 (5.36, 9.43)	16.22 (12.19, 21.59)	
P value	-	<0.001	<0.001	<0.001	<0.001	
**Site of delivery**						
Home	923 (8.0%)	2793 (21.3%)	5319 (36.5%)	9503 (56.1%)	14463 (72.7%)	33001 (43.3%)
Private health centre	1259 (10.9%)	1300 (9.9%)	974 (6.7%)	716 (4.2%)	529 (2.7%)	4778 (6.3%)
Government health centre	2214 (19.2%)	3428 (26.1%)	4098 (28.1%)	3743 (22.1%)	2617 (13.2%)	16100 (21.2%)
District or regional hospital	7122 (61.8%)	5618 (42.8%)	4179 (28.7%)	2988 (17.6%)	2287 (11.5%)	22194 (29.2%)
Total	11518 (100%)	13139 (100%)	14570 (100%)	16950 (100%)	19896 (100%)	76073 (100%)
Adjusted odds ratio (95%CI)***	1.00	3.23 (2.93, 5.84)	6.92 (5.22, 7.95)	9.87 (6.77, 12.95)	17.98 (11.82, 23.90)	
P value	-	<0.001	<0.001	<0.001	<0.001	

Quality of pregnancy care analyses conducted for births in which there had been at least one episode of pregnancy care and no missing data (n = 29 687).

Pregnancy care analyses conducted for births with no missing data (n = 76 129).

Site of delivery analyses conducted for births with no missing data (n = 76 073).

* Odds ratio calculated for the binary outcome comparing those with adequate care (≥2 doses tetanus toxoid) to those with inadequate care (<2 doses tetanus toxoid) by wealth quintile, and adjusting for maternal age, ethnic group, gravidity, previous stillbirth, number of infants delivered, sex of the infant and for clustering by woman.

** Odds ratio calculated for the binary outcome comparing those with any pregnancy care to those with no pregnancy care by wealth quintile, and adjusting for maternal age, ethnic group, gravidity, previous stillbirth, number of infants delivered, sex of the infant and for clustering by woman.

*** Odds ratio calculated for the binary outcome comparing those who delivered at a heath facility to those who delivered at home by wealth quintile, and adjusting for maternal age, ethnic group, gravidity, previous stillbirth, number of infants delivered, sex of the infant and for clustering by woman.

Women also had an increased risk of delivering an antepartum (adjOR 2.59 [2.21, 3.00], p value <0.001) and intrapartum (adjOR 2.22 [1.86, 2.64,] p value <0.001) stillbirth in a district hospital compared to women who delivered at home or in a health centre (Table 2).

### Wealth quintile

There was no association between wealth quintile and antepartum stillbirths in the univariable analysis and the results did not change substantially after adjusting for utilisation of pregnancy care or other sociodemographic variables(adjOR 0.98 [0.93–1.03], p value 0.400) (Table 4). There was also no modification of the effect of health service utilisation on antepartum stillbirth risk by socioeconomic status (likelihood ratio test statistic  = 2.11, p value  = 0.15).

In contrast there was a marked ‘dose response’ of increasing risk of intrapartum stillbirth with increasing levels of socioeconomic deprivation (adjOR 1.08 [1.03–1.14], p value 0.002) (Table 4). Women in the poorest two quintiles had greater risk of intrapartum stillbirth compared to the richest women (adjOR 1.19 [1.04–1.36] p value 0.014). This risk was not substantially altered by adjusting for utilisation of pregnancy care (adjOR 1.19 [1.04–1.37] p value 0.013) or other variables in the multivariable analyses (adjOR 1.19 [1.02–1.38] p value 0.023). Health service utilisation also appeared to reduce intrapartum stillbirths in rich but not poor women. Pregnancy care was associated with a statistically significant decrease in intrapartum stillbirths in women in the richest three quintiles (adjOR 0.87[0.79–0.96] p value 0.007) but there was no effect in women in the poorer two quintiles (adjOR 1.03 [0.95–1.10] p value 0.498), and there was statistical evidence of modification of the effect of health service utilisation on intrapartum stillbirths by socioeconomic status (likelihood ratio test statistic 6.50, p value 0.011).

## Discussion

Poor women had a high risk of intrapartum stillbirth in this rural Ghanaian population and this risk did not appear to be influenced by health service utilisation. In contrast, socioeconomic status was not associated with antepartum stillbirth risk in univariable or multivariable analyses.

To our knowledge this is the first study that has examined socioeconomic differentials in antepartum and intrapartum stillbirth risk in a low or middle income country and in sub-Saharan Africa. There was a marked ‘dose response’ of increasing intrapartum stillbirth with increasing levels of socioeconomic deprivation. Poor women had the highest stillbirth risk and the most limited use of pregnancy care. Pregnancy care also did not appear to influence intrapartum stillbirth risk in the poor women who were able to use health services. This was in contrast to a marked effect in richer women. This may be because poorer women receive suboptimal quality pregnancy care. Pregnancy clinics in the study area should provide interventions that reduce the risk of intrapartum stillbirth such as education about birth preparedness, danger signs, care seeking and facility delivery [8]. We had limited data on quality of care but our study suggested that poor women were more likely to receive suboptimal quality pregnancy care than richer women. In addition, poor women may not be able to use pregnancy services effectively. For example they may not have the resources or money to follow recommendations for care seeking at the time of delivery. There also appeared to be differentials in use of health facilities during labour and delivery. Rich women were more likely to use facilities for delivery compared to poorer women; these differentials were most striking for district hospitals and less striking for the smaller private and government health facilities. However, we had no data on the types of facilities that provided pregnancy care in our study.

We also reported that antepartum stillbirth risk was not affected by socioeconomic status. Other studies from high income countries have described similar findings. [3], [17], [18] These studies have also indicated that the causes of antepartum stillbirth are multifactorial, complex, unrelated to use of health services and may have a genetic component [2], [17]–[19]. However, there have been few studies which have examined these factors in low or middle income countries. We found associations between antepartum stillbirth risk and previous stillbirth, older age group, primiparity and multiple pregnancies. These findings are similar to many other studies from low and middle income countries [1], [20]–[22]. In addition, we reported that pregnancy care was associated with significant reductions in antepartum stillbirth. Pregnancy clinics in Ghana provide interventions that reduce the risk of antepartum stillbirth such as iron and folate supplementation and screening for malnutrition and infection and there have been many improvements in these services in recent years.

There were several limitations to our study. We were only able to use maternal recall to estimate gestational age and we defined a stillbirth as a pregnancy loss resulting after a gestation period of ≥6 months (approximately 26 weeks). A cut off at 28 weeks gestation is commonly used to classify stillbirths in low and middle income countries [1], [7]. Thus, we may have overestimated our stillbirth risk. However, our stillbirth risk (32.4/1000) was similar to recently published data (28.3/1000) for Ghana in 2009 [7]. Our neonatal mortality risk was also similar to previously published studies [23]. Our study was also population based and fieldworkers visited study participants at their homes every four weeks. Migrations were tracked and few women who migrated within the study area were lost to follow up. Thus, only a small proportion of pregnancies and pregnancy outcomes were missed.

**Table 4:** Associations between stillbirths and maternal socioeconomic status, 1 July 2003 to 30 September 2008.

	Antepartum stillbirths			Intrapartum stillbirths		
	Adjusted OR model 1 (95% CI)	Adjusted OR model 2 (95% CI)	Adjusted OR model 3 OR (95% CI)	Adjusted OR model 1 (95% CI)	Adjusted OR model 2 (95% CI)	Adjusted OR model 3 OR (95% CI)
**Wealth quintile**						
Highest (richest)	1.00	1.00	1.00	1.00	1.00	1.00
Second	0.98 (0.79, 1.20)	0.97 (0.79, 1.20)	0.96 (0.79, 1.18)	1.09 (0.84, 1.41)	1.09 (0.84, 1.41)	1.09 (0.84, 1.41)
Middle	0.93 (0.76, 1.15)	0.93 (0.75, 1.14)	0.92 (0.75, 1.13)	1.32 (1.03, 1.69)	1.32 (1.03, 1.69)	1.37 (1.07, 1.75)
Fourth	1.02 (0.84, 1.24)	1.00 (0.82, 1.22)	1.00 (0.82, 1.23)	1.37 (1.07, 1.74)	1.37 (1.08, 1.74)	1.38 (1.08, 1.77)
Lowest (poorest)	0.84 (0.69, 1.03)	0.81 (0.66, 0.99)	0.88 (0.71, 1.09)	1.36 (1.08, 1.72)	1.37 (1.08, 1.73)	1.42 (1.10, 1.83)
P-value	0.24	0.11	0.59	0.02	0.02	0.01
**Linear trend per wealth quintile**	0.97 (0.93, 1.01)	0.96 (0.92, 1.00)	0.98 (0.93, 1.03)	1.08 (1.03, 1.14)	1.08 (1.03, 1.14)	1.09 (1.03, 1.16)
P-value	0.15	0.06	0.37	0.002	0.002	0.002

Analyses conducted on mothers and infants with no missing data (n = 76 129).

Model 1 adjusted for clustering by woman.

Model 2 adjusted for health service utilisation (number of episodes of pregnancy care) and clustering by woman.

Model 3 adjusted for health service utilisation (number of episodes of pregnancy care), maternal age, ethnic group, gravidity, previous stillbirth, number of infants delivered, sex of the infant, and clustering by woman.

Partographs and other tools to estimate fetal movements are not commonly used in our study area [8]. Thus we were only able to classify antepartum and intrapartum stillbirths on the basis of maternal history using a VPM questionnaire. However, the VPM questionnaire included detailed questions about fetal movements before and during labour and also included a comprehensive open history section where the mother retold the story of her pregnancy, labour and delivery in her own words. Each VPM was read by experienced physicians who had to reach agreement before the timing of the death was classified. These methods have previously been validated in our study area and the short period of time between the occurrence of a stillbirth and the VPM is likely to have minimised recall bias [14], [15]. Our ratio of antepartum to intrapartum stillbirths is also consistent with regional estimates [1], [7].

Data were not collected on the use of tobacco and alcohol as these behaviours are known to be infrequent or non-existent in pregnant women in our study area. However, we collected data for other important potential confounders and presented unadjusted and adjusted results. Most of our information was obtained through maternal recall and was not able to be validated from health service records. However, our study was prospective and information about important exposures was obtained during pregnancy before birth outcomes were reported. Thus, unlike many other studies, these data were not influenced by the mother's recall at the time of delivery. We also had limited data on the functioning of the health centres including the quality of care. Our one proxy (tetanus toxoid vaccination) indicated that poor women have suboptimal quality of care compared to richer women but more detailed studies are needed to examine this issue. Finally, this study was conducted in seven contiguous rural districts of the Brong Ahafo region of Ghana. Our results are likely to be generalisable to other rural areas of sub-Saharan Africa. However, further studies are needed from other African settings including urban areas.

This study has important implications for policy and program development. Perinatal health outcomes should not be assumed to be homogeneous within rural areas in low and middle income countries. In our study, poor women had the highest stillbirth burden, the poorest use of pregnancy care and the poorest quality of care. This is yet another example of the inverse care law, i.e. that the availability of good medical care tends to vary inversely with need [24]. Health system strengthening is required to meet the needs of poor women in sub-Saharan Africa. New models of care and innovative delivery channels must be provided. Programmes used in Asia such as home visiting from community health workers, peer counselling and women's groups could be highly effective in meeting the needs of poor African women with limited access to conventional pregnancy care. Data from the implementation and evaluation of these programmes in sub-Saharan Africa are greatly needed.
